# Is traditional male circumcision effective as an HIV prevention strategy? Evidence from Lesotho

**DOI:** 10.1371/journal.pone.0177076

**Published:** 2017-05-12

**Authors:** Elisa M. Maffioli

**Affiliations:** 1 Department of Economics, Duke University Durham, NC, United States of America; 2 Duke Global Health Institute, Duke University Durham, NC, United States of America; University of Ottawa, CANADA

## Abstract

In many developing countries, male circumcision has been promoted as an effective HIV prevention strategy, and medical randomized controlled trials have indeed shown a causal link. However, there is limited empirical evidence to support this conclusion in countries where individuals can voluntary opt for different types of circumcision. The present study considers male circumcision in Lesotho, where HIV prevalence is among the highest in the world (23%). Here, men can opt for one of two types of circumcision: traditional male circumcision in initiation schools, or the medical option in health clinics. This paper investigates whether the former has medical effects on individual HIV status that are as beneficial as those shown for the latter. Controlling for the potential individual behavioral response after the operation, it was found that circumcision performed in initiation schools wholly offset the medical benefits of the surgical procedure. This supports anecdotal evidence that the operation performed by traditional circumcisers does not have the same protective effect against HIV transmission as the medical operation. No evidence of “disinhibition” behavior among circumcised men was found, nor differential risky sexual behavior among men circumcised, traditionally or medically. Considering that, in Lesotho, traditional male circumcision is undertaken by more than 90% of circumcised men, the findings highlight the need for further research into how the operation in initiation schools is performed and its medical benefits.

## Introduction

Sub-Saharan Africa is the region most severely impacted by HIV/AIDS in the world. It has been estimated that 25.8 million people live with HIV, and 1.4 million were newly infected in 2014 [1]. Evidence from three randomized controlled trials (RCTs) showed that Voluntary Medical Male Circumcision (VMMC) reduced the chances of female-to-male HIV transmission by between 48 and 60% [2–4]. Following these results, VMMC was promoted as an effective HIV prevention strategy in many African countries [5]. Yet, in Sub-Saharan Africa, the most common type of circumcision is not performed in a clean or safe setting by trained health professionals. Most men are still circumcised traditionally (TMC), often in initiation schools during the rite of passage to manhood. This type of surgical procedure frequently takes place in non-clinical environments, in poor hygienic conditions, where practitioners have different types of knowledge and training when compared to western-trained doctors or they are not equipped with proper supplies. It has been empirically demonstrated that the risk of adverse events, including medical complications and death, is significantly higher in traditional rites than for surgical operations performed in medical settings [6–8].

Male circumcision (MC) is one of the oldest known surgical procedures, traditionally performed as a mark of cultural identity or religious observance. Circumcision was widely practiced among ancient Semitic peoples, including Egyptians and Jews. Muslims are the largest religious group to practice MC as a confirmation of their relationship with God. The practice is closely associated with ethnicity in Sub-Saharan Africa, where it has been performed for non-religious reasons for thousands of years; still today, it is an integral part of a rite of passage to manhood [9]. In many African countries, VMMC has become important as an effective HIV prevention strategy. Governments, supported by international organizations such as UNAIDS and the WHO, started extensive campaigns to promote VMMC as part of HIV prevention strategies. In Lesotho, where HIV prevalence in adults aged 15 to 49 is 23.4% [10] (the second highest in the world), a national circumcision program was launched in 2012 to combat it. In more developed countries, and the United States in particular, the procedure was introduced around the 20th century for health-related and social reasons. Although widely practiced, it is not currently medically sanctioned. It is also not generally associated with religion or ethnicity.

MC consists of the surgical removal of some or all of the foreskin (prepuce) from the penis. In uncircumcised men, the area under the foreskin–and the foreskin itself–contains many of the cells the virus binds to (the Langerhans’ cells) and the skin or mucosal surface of the foreskin is more easily penetrated by the virus [11–12]. Circumcised men, due to the removal of these cells through the surgical procedure, are less likely to pass the HIV virus and other STIs to female partners [2–4].

Approximately 55% of Basotho men are circumcised [13], and an estimated 15,000 circumcisions are performed every year. This includes two types of circumcisions: medical circumcisions, which take place in authorized health facilities, and traditional operations performed in initiation schools during the traditional rite of “lebollo”. Two-thirds of all circumcisions in Lesotho fall under this category [13]. Most of the Basotho men who undergo the latter procedure are circumcised in late adolescence (16–20 years), while the majority of males who seek circumcision in health facilities are adults. Very few parents bring their infants (0–2 years) to hospitals to undertake safe circumcisions, despite that, at this age, the procedure is much simpler and safer [14].

Depending on how the surgical operation is performed, MC may not be protective in the prevention of HIV transmission. TMC, for example, does not often involve a complete removal of the foreskin, but is more of a symbolic cut. Misreporting of MC is also common. Basotho men often believe they are circumcised according to their own definition, which might be different from the medical one. In a RCT performed in South Africa [3], researchers found that 59% of men reported that they were circumcised, but, upon inspection, doctors found that the entire foreskin was not removed. A recent study of Lesotho Defence Force applicants showed that half of the men who reported that they were circumcised retained all or a portion of their foreskin, as assessed through a physical exam [15]. The proportion of men who had had their foreskin completely removed was significantly higher among those who had been medically circumcised than those who had been circumcised in an initiation school. Since the surgical procedure might differ depending upon where and who performs it, TMC may not afford the same degree of protection in terms of HIV transmission as VMMC. Quantitative and qualitative studies, supported by anecdotal evidence, cast doubt on the effectiveness of MC performed in initiation schools, compared to the safe procedure that takes place in health facilities. It is unclear whether the former type of MC may also be damaging to the individuals involved. This study aims to fill this gap in knowledge by analyzing the relationship between TMC and individual HIV status.

Causal evidence provided by RCTs [2–4], which showed that VMMC is effective in preventing HIV transmission, was fundamental in advancing medical research. However, it did not provide a satisfying picture for the promotion of both types of MC as HIV prevention strategies. The comparison with the general population is questionable given the different types of procedures performed, and the conditions under which the RCTs were conducted. First, the VMMC performed in the experimental settings was found to be a different surgical procedure than TMC. In countries like Lesotho, where 91% of the circumcised men underwent circumcision in initiation schools rather than in health facilities, the results from experimental interventions are difficult to generalize to the entire population. Second, in experimental settings, study participants received a package of benefits including counseling, free condoms, and, in some cases, financial incentives to undergo VMMC. The researchers argued that VMMC was associated with lower risk of HIV transmission, and did not find evidence of risk-compensation behavior after MC [16]. In population studies, however, results might differ, both because of the two types of circumcision men can choose between, and because of the lack of counseling associated with the surgical procedure. Information campaigns promoting the message that MC reduces the risk of HIV transmission, without pointing out that only VMMC has beneficial medical effects, can promote a false sense of immunity. This might increase the chance that circumcised men will engage in risky sexual behavior. Individuals who believe MC to be effective in preventing HIV transmission may, consequently, increase their risk for HIV. Thus, the increased propensity for risky sexual behavior among circumcised men can overcome the medical benefits of the surgical operation per se.

At first glance, Lesotho is one of the few countries in which HIV prevalence is higher among circumcised men than among uncircumcised men (Demographic Health Surveys 2004 [22.8% vs 14.2%] and 2009 [21% vs 16%]) [13]. Prior studies using national surveys found no association between individual HIV status and MC [17]. However, the relationship between HIV status and MC could differ depending on the type of MC performed. Using the Demographic Health Survey 2009 (DHS) [13], the present study examined a similar association. Since little is known in Lesotho about how and by whom the surgical operations in initiation schools are performed or their potential medical consequences, this study focuses on TMC.

There are two primary mechanisms that may underlie the association between MC and individual HIV status, and which prevails could differ between the two types of MC. The first is the type of surgical procedure performed. If the procedure is performed in a medical setting and consists (as is likely) of the complete removal of the foreskin, the association between VMMC and HIV status should be negative, as confirmed by experimental evidence [2–4]. On the other hand, given the uncertainty about how the surgical procedure is performed in initiation schools and whether young boys are assisted by health professionals during the operation, the association between TMC and HIV positive status remains an open empirical question.

The second is the individual behavior response. If circumcised men believe MC to reduce the probability of their contracting HIV, they may feel that they are safer from or even immune to HIV, and engage in riskier sexual behavior. This type of reaction, called “disinhibition behavior” or risk compensation behavior, was found to exist in similar settings, such as after increased access to ART availability in Sub-Saharan Africa [18, 19] and after being found HIV negative [20]. Several studies have explored behavioral responses to beliefs about HIV status [21], information about HIV [22], or information about MC [23–25], but evidence is still scarce on the behavioral response to the surgical operation of MC per se. Experimental studies have examined the behavioral response to MC [2–4, 16, 26], finding mixed evidence about differences in risky sexual behavior between circumcised and uncircumcised men. Still, little evidence exists when comparing sexual behavior among medically or traditionally circumcised individuals [27]. In the absence of risk compensation behavior, we expect VMMC to be more effective than TMC in preventing HIV transmission. If risk compensation among circumcised men exists, we expect this behavior to be stronger in cases of VMMC than TMC, since medically circumcised men are more likely to receive counseling about the surgical procedure and the medical benefits associated with it, compared to traditionally circumcised men.

The analysis focuses on understanding how the surgical procedure performed in initiation schools is associated with individual HIV status, and to what extent a potential individual behavioral response to TMC may offset the (unknown) medical benefits in a real-world setting. Even though the analysis controls for many potential confounders, we cannot argue to fully identify a causal relationship. However, understanding both the risks and advantages of performing MC in initiation schools compared to health facilities is crucial in implementing appropriate interventions. In the past, public campaigns mainly focused on VMMC, but there is a lack of knowledge about whether and to what extent TMC is effective as an HIV prevention strategy compared to the better-studied and advertised VMMC.

## Methods

This study used data from the Demographic and Health Survey (DHS) 2009 [13]. The fieldwork was implemented between October 2009 and January 2010, with the support of the Ministry of Health and Social Welfare (MOHSW): 9,994 households were sampled, and 9,391 of these households participated in the survey. Among them, 3,493 men were identified as eligible for the male questionnaire, because they were between 15 and 59 years old. Of these men, 3,317 agreed to participate in the individual interview. We used information from the survey about the respondents’ background, reproduction, marriage and sexual activity, employment, HIV/AIDS status, and MC-related questions. We restricted the sample to 2,735 sexually active men for the analysis. Since the potential beneficial effect of MC on HIV status operates by reducing HIV transmission during sexual contacts, we excluded men who reported that they had never had sexual intercourse in their lifetime.

The sample was then merged with the 2009 AIDS Indicator Survey (DHS), in which respondents’ HIV status was recorded. In Lesotho, the DHS testing protocol provides anonymous, informed, and voluntary testing. The protocol consists of collecting blood spots on filter paper from a finger prick and transporting them to a laboratory for testing. An initial ELISA test (Enzyme-Linked Immune-Sorbent Assay) was performed in a laboratory and, after that, a retesting of all positive tests and 5–10 percent of the negative tests with a second ELISA followed. For those with discordant results on the two ELISA tests, a new ELISA or a Western Blot test was necessary. Every respondent received educational materials and referrals for free testing and counseling. Since the population-based testing was dependent on the population’s willingness to be tested for HIV, in those cases where the characteristics of those who agreed to be tested differed from those who refused, a potential bias may have resulted. 189 men refused to be tested for HIV; of these, 110 were circumcised. T-tests for the means were used to determine any significant difference (*p*<0.05) in socio-demographic characteristics and risky sexual behavior among sexually active men who agreed or refused to be tested for HIV. Men who refused to be tested had higher levels of education, were less likely to live in rural areas, were slightly older, and were more likely to be married. They were also less likely to be employed in agriculture, and more likely to work regularly. As far as their behavior was concerned, based on several indicators of risky sexual activities (Table 1), men who refused to be tested for HIV appeared to be less sexually risky in term of having extramarital relations and their age of first sexual encounter. Since the study describes the association between MC, risky sexual behavior, and HIV status, concerns can be raised about differences in the sub-sample of circumcised men who decided to be tested for HIV. Very few statistically significant differences existed, and were not reported. The empirical specification controlled for all these differences. The final sample for the empirical analysis consisted of 2,546 sexually active men tested for HIV.

**Table 1:** Mean effect analysis: List of variables.

Index	Main variables
1. First experiences	Age at first marriage
	Age at first sexual debut
2. Risky sexual activities	Condom not always used with last partner
	Had extra-marital relationship in the past year
	Sexually active in the last 4 weeks
	Engaged in transactional sex in the past year
	Alcohol used during last sex
3. Number of relationships	No. people had sex in the past year (including wife)
	No. partners in lifetime

The index of “first experiences” is constructed on the sample of ever married individuals. The index of “risky sexual activities” is constructed on the sample of currently married individuals. The index of “number of relationships” is constructed on the full sample in the analysis (sexually active individuals).

The first variable of interest in the analysis was male circumcision (MC), constructed as a dummy equal to 1 if the person answered yes to the question “Are you circumcised?” and 0 otherwise. As previous literature has described [15], the definition may raise concerns, because self-reported measures might not correspond to real status upon physical inspection. We relied on individual circumcision data to identify two types of surgical procedures. We defined medical male circumcision (VMMC) as the surgical procedure that individuals reported to have taken place in health clinics or at the home of a health worker, and we defined traditional male circumcision (TMC) as an operation performed in an initiation school. A different question in the survey collected information about whether MC was performed by a health professional. In almost every case in which the respondent reported that a health professional had performed the surgical procedure, the type of operation was VMMC. Very few were TMC. Even though we cannot exclude that some traditional circumcisers could have been trained in surgical methods comparable to those for VMMC, this fact indicates that TMC is not performed by health professionals in most instances. Thus, even if not perfect, the definitions of the two types of MC based on the location of the surgical procedure adequately distinguish the types of procedures for the scope of the analysis. The main dependent variable relied on HIV status as reported in the AIDS survey, and it was constructed as an indicator for whether the respondent had a positive HIV test result at the time of the survey.

To account for differences among circumcised and uncircumcised men, and men who opted for MC in an initiation school or at a health clinic, basic control variables were added to the empirical specification. The model controlled for respondent socio-demographic characteristics such as age, level of education, employment status, job type, marital status, household size, whether the respondent lived in a rural or urban area, and whether he was poor. Even though the population of Lesotho is estimated to be approximately 90% Christian, different types of Christianity are present. We included indicator variables for Catholic or Protestant Christianity, and we created another category for individuals from other or no religion. Since the ethnic-linguistic structure is comprised of 99.7% Basotho Bantu-speaking people, ethnicity was not part of the set of control variables.

In a country like Lesotho, where the majority of men migrate to South Africa or to other nearby countries to work, especially in the mining sector, migration can be a key determinant for both risky sexual behavior and individual HIV status. Today, Lesotho’s economy continues to depend quite significantly on migrants, with more than 40,000 Basotho migrating every year. Migration is often considered one of the important explanatory elements in why HIV/AIDS is so diffused. We added two variables in the analysis to account for migration as a potential confounding factor, such as in how many separate occasions the respondent traveled and slept away from the home community in the last 12 months, and, if he did travel in the past year, whether the respondent was away continuously for more than one month. Finally, the model included ten district dummies. In the preferred specification, we also included measures of risky sexual behavior. Since several indicators are available in the DHS data, Table 1 describes the selected variables. Following previous studies [28], we used the mean effect analysis to construct single indexes of related outcomes. We summarized them in three main indicators.

The first indicator described the individual’s first sexual experiences: age at first marriage and age at first sexual experience. The second index summarized risky sexual activities: whether a condom was used during the most recent intercourse; whether the respondent had had an extramarital relationship in the past year; whether he was sexually active in the past 4 weeks; whether he had ever engaged in transactional sex; and whether he had used alcohol during the most recent intercourse. The last indicator grouped two variables referring to the number of sexual relationships: the total number of people the man had had sex with in the last year, and the total number of partners in his lifetime. Note that, once summarized in a unique indicator, the first index entailed the sample of individuals who had ever been married; the second was constructed of the sample of currently married men; and the third index summarized risky behavior for the whole sample of sexually active men. After grouping the outcomes, we standardized them by subtracting the mean and dividing by the standard deviation. We then summed the standardized value of the outcomes, dividing it for the grouping’s size (i.e., the number of variables considered in the group). More formally, denoting the grouping of related outcomes by *Y*_*k*_, with *k* = 1, …, *K* being the number of variables in the group, we first defined the standardized outcome variables as $Y_{k}^{*}=\frac{Y_{k}-\mu \left(Y_{k}\right)}{\sigma (Y_{k})}$ with *μ*(*Y*_*k*_) and *σ*(*Y*_*k*_) being respectively the mean and standard deviation of each variable in the group. We then formed a single index given by $\sum_{k}\frac{Y_{k}^{*}}{K}$.

The association between the types of MC and the positive HIV status for an individual *i* in district *d* were analyzed using the following standard logit model:

$$Pr\left(y_{id}=1|X_{id},Z_{id}\right)=\Lambda \left(\beta MC_{id}+\gamma MC_{id}*TMC_{id}+\delta X_{id}+\phi Z_{id}+\psi_{d}\right)$$ (1)

where *y*_*id*_ is expressed as a dummy equals 1 if the respondent was tested positive and 0 if negative. Λ() is the logistic function of the linear regression expression in brackets, where *MC*_*id*_ is a dummy that takes value 1 if the man reported to be circumcised, while *MC*_id_*TMC_*id*_ is an interaction term between being circumcised and a dummy indicating that MC was performed traditionally in initiation school rather than in a health clinic (*TMC*_*id*_); *X*_*id*_ includes all the socio-economic controls, while *Z*_*id*_ is a vector of risky behavior measures. *ψ*_*d*_ refers to the 10 district fixed effects. Standard errors are clustered at the level of the smallest enumeration area for a total of 400 clusters. The coefficient of interest is *γ* that provides the additional effect of being circumcised during initiation to manhood rather than being medically circumcised. Estimates are expressed in Odds Ratio.

## Results

### Description of the sample

The sample of sexually active men was included in the analysis for a total of 2,735 men between 15 and 59 years old (Table 2). The sample was composed of men who were, on average, 31 years old, not highly educated (34% have a secondary or higher level), and mainly employed in agriculture (37%). Most of the sample lived in rural areas (78%), and 42% were defined as poor (based on the first quantiles of DHS wealth index distribution). About half of the sample was currently married or living with a partner. All men were of Basotho ethnicity, and the majority practiced a Christian religion (41% Catholic, 48% Protestant). Migration was also common in the sample, with men who traveled away from the home community on average 3.5 times in the past year, and 23% of them stayed away for more than one month (Table 2, Panel A).

**Table 2:** Respondents’ characteristics.

	Mean	SD [Min-Max]	No. Yes Responses	No. Obs
**Panel A: Demographic Characteristics**				
Age	30.82	11.91 [15–59]		2,735
Educ secondary or higher	34.29		938	2,735
Rural area	77.91		2,131	2,735
Poor	41.61		1,138	2,735
Self-employed in agriculture	37.03		1,004	2,711
Working yearly	34.04		931	2,735
Catholic	41.06		1,123	2,735
Protestant	48.23		1,319	2,735
Married (ever)	54.37		1,487	2,735
Married (currently)	47.38		1,296	2,735
Household size	5.61	2.9 [1–21]		2,735
No. times away in past year	3.47	6.2 [0–29]		2,735
Away in past month	23.03		2,735	2,735
**Panel B: Male circumcision**				
Circumcised	60.99		1,668	2,735
MC by health professional	7.87		131	1,664
Circumcised at initiation school (TMC)	91.42		1,525	1,668
Circumcised at health clinic (VMMC)	8.57		143	1,668
Age at TMC	18.28	3.76[0–54]		1,500
Age at VMMC	18.71	9.69[0–55]		140
**Panel C: Risky behavior**				
Age at first marriage	23.76	4.57[10–49]		1,482
Age at first sexual debut	17.71	3.9[8–40]		2,735
Condom not used in last intercourse	56.05		1,325	2,364
Any extra-marital relationships in the past year	29.32		380	2,735
Sexually active in the last 4 weeks	50.78		1,369	2,696
Engaged in transactional sex in the past year	2.69		72	2,673
Alcohol used during last sex	9.27		250	2,696
No. people had sex in the past year (including wife)	1.18	0.95 [0–26]		2,696
No. partners in lifetime	7.77	13.42 [1–95]		2,598
**Panel C: HIV positive**				
Individual is HIV positive	18.85		480	2,546

The sample consists of all sexually active male individuals, defined as any man who had ever had sexual intercourse. Mean is defined as percentage for discrete variables and as average for continuous variables. Standard deviation (SD) and range [Minimum–Maximum] are included for continuous variables.

Turning to MC (Table 2, Panel B), almost 61% of the sexually active men in the sample were circumcised: 91% of them were circumcised in initiation schools rather than in health facilities or at the home of a health professional. The average age for MC was very similar between the two types of MC, at 18 years. However, the majority of the men who underwent the surgical procedure in initiation schools were circumcised between 15 and 22 years, while men circumcised in health facilities usually underwent MC at younger or older ages (between 3 and 30 years old at the 10th and 90th percentile of the distribution). The different age distributions of the two types of MC is a clear indication that TMC is usually performed during the initiation rite to manhood in adolescence, as part of Basotho culture. In addition, the fact that only 7.8% of the surgical procedures were performed by a health professional, and most of them were VMMC, also supports anecdotal evidence that TMC in Lesotho is not as safe as VMMC.

In term of risky sexual behavior (Table 2, Panel C), men had their first sexual encounter when they were on average 18 years old, but they got married a few years later, around 24 years old. About half of the men in the sample were sexually active in the past month, and more than 56% did not use condoms during the most recent sexual intercourse. It is striking that more than 29% of the currently married individuals had at least one extramarital relationship in the past year. Transactional sex and alcohol use during last sex were not very common. Finally, about 19% of the sexually active men in the sample tested positive for HIV (Table 2, Panel D).

### Sample characteristics by status and type of male circumcision

Table 3 shows the t-test for the differences in demographic characteristics, sexual behavior, and HIV status among circumcised and uncircumcised men, and among the two types of MC. Columns (3) and (6) report p-values for the hypothesis testing of equality of the means. In terms of socio-demographic characteristics (Table 3, Panel A), circumcised individuals were older, less educated, mainly from rural areas, poorer and with a bigger household size, compared to uncircumcised men. They were also more likely to be employed in agriculture or to migrate (*p*<0.05). These characteristics were very similar to the traits that differentiated men who underwent VMMC compared to TMC (column 4 and 5). The only exception was the individuals’ age, since traditionally circumcised men were younger overall compared to men who had undergone VMMC (*p* = 0.049), confirming the cultural practice associated with TMC. Circumcised men (and men who had undergone TMC) were also married at younger ages compared to uncircumcised men (and men who had undergone VMMC) (*p*<0.001). As far as risky behavior was concerned (Table 3, Panel B), circumcised men were also more likely to engage in other risky activities than uncircumcised men. We found statistically significant differences in whether they had had any extramarital relationships (*p* = 0.015), whether they were sexually active in the past 4 weeks (*p* = 0.002), the number of people they had sex with in past year (*p* = 0.012), and condom (*p*<0.001) and alcohol use (*p* = 0.079) during past intercourse. On the other hand, traditionally circumcised men were less likely to be sexually active in the past month (*p* = 0.001), and had a lower number of partners over their lifetimes (*p* = 0.007) compared to medically circumcised men, but they were less likely to use condoms in the past intercourse (*p*<0.001). Panel C (Table 3) describes the percentages of HIV positive individuals for each category. Circumcised men were more likely to be HIV positive than uncircumcised men (19.64% and 17.61% respectively), and traditionally circumcised men were also more likely to be HIV positive than men who had undergone VMMC (20.07% and 14.84% respectively). Differences are not statistically significant at 10% level.

**Table 3:** Respondents’ characteristics by male circumcision status and type of circumcision.

	Not circumcised	Circumcised		VMMC	TMC	
	Mean (SD)	Mean (SD)	pvalue	Mean (SD)	Mean (SD)	pvalue
	(1)	(2)	(3)	(4)	(5)	(6)
**Panel A: Demographic characteristics**						
Age	29.27 (12.26)	31.82 (11.58)	<0.001	33.64 (11.34)	31.65 (11.59)	0.049
Educ secondary or higher	53.33	22.12	<0.001	81.12	16.59	<0.001
Rural area	65.89	85.61	<0.001	34.97	90.63	<0.001
Poor	26.05	51.56	<0.001	13.29	55.15	<0.001
Self-employed in agriculture	25.24	44.54	<0.001	11.97	47.59	<0.001
Working yearly	35.61	33.03	0.165	54.55	31.02	<0.001
Catholic	42.92	39.87	0.113	41.96	39.67	0.594
Protestant	48.27	48.2	0.974	41.96	48.79	0.118
Married (ever)	44.33	60.79	<0.001	67.13	60.2	0.104
Married (currently)	38.89	52.82	<0.001	57.34	52.39	0.257
Household size	5.33 (2.82)	5.8 (2.96)	<0.001	4.45 (2.43)	5.92 (2.97)	<0.001
No. times away in past year	3.71 (6.4)	3.31 (6.08)	0.103	5.97 (8.95)	3.06 (5.67)	<0.001
Away in past month	20.15	24.88	0.004	24.48	24.92	0.907
**Panel B: Risky behavior**						
Age at first marriage	24.64 (4.82)	23.35 (4.39)	<0.001	25.2 (4.59)	23.16 (4.33)	<0.001
Age at first sexual debut	17.39 (3.95)	17.92 (3.86)	0.001	18.08 (3.75)	17.9 (3.87)	0.589
Condom not used in last intercourse	43.94	63.27	<0.001	48.06	64.72	<0.001
Any extra-marital relationships in past year	24.82	31.44	0.015	24.39	31.17	0.149
Sexually active in the last 4 weeks	46.7	53.18	0.002	67.14	51.89	0.001
Engaged in transactional sex in the past year	2.12	3.05	0.148	1.46	3.2	0.257
Alcohol used during last sex	8.04	10.05	0.079	9.29	10.13	0.752
No. partners in lifetime	7.26 (13.46)	8.09 (13.39)	0.126	11.04 (17.74)	7.82 (12.88)	0.007
No. people had sex in the past year (including wife)	1.13 (0.90)	1.22 (0.97)	0.012	1.36 (0.84)	1.22 (0.98)	0.588
**Panel C: HIV positive**						
Individual is HIV positive	17.61	19.64	0.202	14.84	20.07	0.154

The sample consists of all sexually active male individuals, defined as any man who had ever had sexual intercourse. Mean is defined as percentage for discrete variables and as average for continuous variables. Standard deviation (SD) is included for continuous variables.

### Main results

Table 4 outlines the association between MC, and in particular TMC, and individual HIV status. Column (1) and (3) replicated the lack of (un-adjusted) statistically significant differences in HIV positive status between circumcised and uncircumcised men, and men who had undergone VMMC or TMC described in Table 3, Panel C. Adjusting the model for district-fixed effects and for socio-demographic, migration, and religion controls, Column (2) confirmed the lack of association between MC and HIV positive status (OR 0.99, CI: 0.78—1.26, *p* = 0.933), as found in other studies [17]. Columns 3–9 explored how different types of MC were associated with individual HIV status. Controlling for district fixed effects and several potential confounders, estimates in Column 5 showed that men circumcised in health clinics were less likely to be HIV positive (OR 0.49, CI: 0.28—0.86, *p* = 0.013). However, the medically beneficial effects of VMMC were balanced-off when the surgical operation was performed in initiation schools. Traditionally circumcised men were indeed more likely to be HIV positive compared to medically circumcised men (OR 2.31, CI: 1.25—4.24, *p* = 0.007).

**Table 4:** Male circumcision and HIV status.

Dependent variable: HIV positive status	(1)	(2)	(3)	(4)	(5)	(6)	(7)	(8)	(9)
Circumcised	1.1434	0.9897	0.8155	0.8753	0.4876**	0.3949***	0.3077***	0.5015**	0.4671**
	(0.119)	(0.122)	(0.203)	(0.222)	(0.141)	(0.129)	(0.127)	(0.151)	(0.150)
Circumcised X TMC			1.4405	1.4300	2.3071***	2.7907***	3.5355***	2.0708**	2.2625**
			(0.364)	(0.373)	(0.716)	(0.966)	(1.528)	(0.655)	(0.754)
Index of first experiences						0.8658*			
						(0.074)			
Index of risky behavior							1.0697		
							(0.200)		
Index of relationships								1.1583*	
								(0.102)	
Condom not used in last intercourse									0.5548***
									(0.080)
Sexually active in the last 4 weeks									1.0623
									(0.153)
No. partners in lifetime									1.0106***
									(0.004)
District FE		✓		✓	✓	✓	✓	✓	✓
Controls		✓			✓	✓	✓	✓	✓
Observations	2,546	2,526	2,546	2,546	2,526	1,350	1,114	2,393	2,083

*** p<0.01, ** p<0.05, * p<0.1. Standard errors in parentheses. The main specification is a Logistic model. Odd Ratios are reported. Controls include socio-economic factors: age and age squared of the respondent; a dummy for secondary or higher education; whether the respondent lives in rural areas; whether he is married or living with a partner; whether he is in the two lowest quantiles of the income distribution as defined by the DHS wealth index; whether he is self-employed in agriculture or working yearly; and household size. Migration variables: on how many separate occasions the respondent traveled away from the home community and slept away in the last 12 months, and whether the respondent was away from the home community for more than one month continuously; dummies for whether the respondent is Catholic, Protestant or from other/no religion. District fixed effects are also included. Standard errors are clustered at the smallest enumeration area.

The estimates could reflect the fact that the surgical operation of TMC did not have the same beneficial medical effects as VMMC, or the fact that traditionally and medically circumcised men had different levels of risky sexual behavior after MC. Each of the two mechanisms separately could explain the evidence that traditionally circumcised men were more likely to be HIV positive than medically circumcised men. Columns (6) to (9) attempt to control for differences in individual risky sexual behavior, using both the three constructed indexes of risky behavior (columns 6–8), and a sub-set of indicators (column 9). Adjusting for potential compensation behavior, estimates of the association between MC and TMC and HIV positive status remained robust (columns 6–8). Results were similar in column 9, which included a sub-set of single indicators of risky sexual behavior, chosen as the best fit by performing likelihood ratio tests and comparing Akaike information criterion [29]. Having a high number of lifetime partners slightly increased the chances of being HIV positive (OR 1.01, CI = 1.00 1.02, *p* = 0.03). Not using a condom in the most recent sexual intercourse was negatively associated with individual HIV status (OR = 0.55, CI:0.42-0.73, *p*<0.001). This pattern could be driven by the habit of not using a condom with long-term partners (72% of individuals in the sample did not use condoms if married). Overall, the results suggest that risky sexual behavior is not the major driver of the relationship between TMC and HIV status, but the type of surgical operation could likely be. The results show that traditionally circumcised men were more likely to be HIV positive compared to men who underwent VMMC. Adjusting for potential risk compensation behavior, TMC did not exhibit the same medically beneficial effect as VMMC.

Table 5 formally tests the association between MC and, in particular, TMC, and “disinhibition behavior”. Given the robustness of the estimates in Table 4 when controlling for risky behavior, we expect a lack of association between the type of MC and indicators of risky sexual activities. If anything, assuming that misinformation is more common among less educated men (i.e., men circumcised in initiation schools), we could expect higher propensity for risky sexual behavior in this sub-sample. Results showed a lack of statistically significant association between the two types of MC and summary indexes of risky sexual behavior. S1 Table presents a dis-aggregated analysis of each of the indicators, and it provides evidence of statistically significant associations for few risky behavior variables. Circumcised men in medical settings were more likely to be sexually active compared to uncircumcised men, while traditionally circumcised men got married earlier compared to medically circumcised individuals. Overall, the estimates did not provide any strong evidence of risk compensation behavior among circumcised men. We also did not find differential risky sexual behavior among men who had undergone VMMC or TMC.

**Table 5:** Male circumcision and indexes of risky sexual behavior.

Dependent variable:	First experiences	Risky behavior	No. relationships
	(1)	(2)	(3)
Circumcised	-0.0276	0.0247	0.0835
	(0.081)	(0.064)	(0.077)
Circumcised X TMC	-0.1212	0.0315	0.0309
	(0.079)	(0.067)	(0.082)
Age	0.1006***	0.0102	0.0296***
	(0.013)	(0.009)	(0.010)
Age squared	-0.0009***	-0.0001	-0.0003**
	(0.000)	(0.000)	(0.000)
Educ secondary or higher	0.0385	-0.0116	0.1301***
	(0.047)	(0.036)	(0.043)
Rural area	0.0463	0.0245	-0.0453
	(0.060)	(0.038)	(0.050)
Married	0.0389		0.0369
	(0.064)		(0.045)
Poor	0.0963*	0.1024**	-0.0383
	Q(0.050)	(0.040)	(0.033)
Self-employed in agriculture	-0.0073	-0.0048	-0.0283
	(0.046)	(0.032)	(0.037)
Working yearly	0.0970**	0.0487*	0.0195
	(0.042)	(0.029)	(0.031)
Household size	-0.0051	-0.0042	-0.0041
	(0.006)	(0.005)	(0.005)
No. times away in past year	-0.0033	-0.0000	0.0045*
	(0.003)	(0.002)	(0.003)
Away in past month	0.0170	0.0275	0.0183
	(0.046)	(0.032)	(0.033)
District FE	✓	✓	✓
Religion controls	✓	✓	✓
Observations	1,469	1,211	2,550

*** p<0.01, ** p<0.05, * p<0.1. Standard errors in parentheses. The main specification is an Ordinary Least Square (OLS). Religion controls include dummies for whether the respondent is Catholic, Protestant, or from other/no religion. District fixed effects are also included. Standard errors are clustered at the smallest enumeration area.

## Discussion

This paper examines the relationship between MC–and traditional male circumcision in particular–and individual HIV status in Lesotho. The analysis builds upon strong evidence provided by three randomized controlled trials conducted in South Africa, Uganda, and Kenya between 2004 and 2007, which showed significant and positive medical effects of VMMC in reducing the risk of female-to-male HIV transmission. The results, however, were based on experimental interventions, and focused on medical male circumcision performed in safe, clean settings. It was, therefore, critical to conduct an additional empirical analysis that makes use of a nationally representative survey to investigate whether the same conclusion can be extended to real-world settings, where men can freely choose to be circumcised in initiation schools (TMC) or at health facilities (VMMC). This analysis is vital for Lesotho, which has one of the highest rates of HIV in the world, and where the vast majority of men are still circumcised in the traditional way.

This study considered the association between individual HIV status and the two existing types of MC in the country, in order to understand whether different types of MC are differently associated with individual HIV status. When studying the association between MC and HIV status, two opposing mechanisms must be considered: a medical channel, which leads to a reduction in the risk of contracting HIV due to the physical surgical procedure, and an individual behavioral channel, which may increase the probability of HIV infection because of engagement in riskier sexual behavior following MC.

Controlling for risky sexual behavior, migration, and several socio-economic characteristics that differentiate men who opt for either type of MC or none, medically circumcised men were found to be less likely to be infected by HIV than uncircumcised men. Consistent with results in experimental settings [2–4], this finding confirmed that VMMC also had beneficial medical effects in the general population in the real-world setting of Lesotho.

It was also found that this negative association was completely offset for individuals who had undergone TMC. This is the most important finding of the study, since it suggests that this type of operation does not have any medical benefits like VMMC. Additional analysis of the association between MC and risky sexual activities indicated that individuals did not engage in riskier sexual activities following any of the two types of MC. The results are in line with past studies, which did not find any evidence of risk compensation behavior among men who had undergone VMMC [16]. In addition, this study also found that men who had undergone TMC did not engage in riskier sexual activities after the surgical procedure. The latter finding is especially important given the lack of information and counseling surrounding this type of procedure.

Taken together, the results emphasize that risk compensation behavior does not play a role in explaining the associations between TMC and HIV status. Instead, the type of surgical procedure performed in initiation schools could help explain the different associations between TMC and VMMC, and HIV status.

The study has some limitations. First, the results represent associations rather than a causal relationship. We attempted to control for as many confounding variables as possible, without over-fitting the empirical model. However, there are still valid concerns about other, unaddressed variables, such as prior beliefs on the personal risk of contracting HIV. Second, the measures of MC used in the analysis are self-reported, and individuals might not have accurate knowledge about how the surgical operation should be performed. The analysis relies on information about the location of MC procedures to distinguish between the two types of MC. Even though this is certainly not always accurate, we do not have any better data to infer the type of surgical procedure performed at each location. Finally, the study cannot describe any temporal relationship, since we do not have information about whether the subjects were circumcised before or after contracting HIV. While the men who underwent VMMC had to have been HIV-negative to be eligible for the surgical procedure, this is not the case for TMC. The data do not provide any information on whether men who had undergone TMC were already HIV positive at the time of circumcision. If that were the case, TMC could not have any beneficial medical effects on individual HIV status, despite the surgical procedure being performed correctly to prevent HIV transmission.

Despite these limitations, this study may be of particular interest to policymakers and governments who are widely promoting MC as one of the most effective HIV prevention strategies. There is a general understanding that MC can reduce the risk of contracting HIV. However, in Lesotho, where the operation is primarily performed in initiation schools, information campaigns can be important in promoting the proper type of circumcision: VMMC. Indeed, the analysis showed that MC performed in clinical settings had medically beneficial effects in reducing HIV transmission, but the same conclusion could not be drawn for TMC. Further research on the latter type of (traditional) surgical procedure and its potentially adverse consequences would be very informative, given the limited knowledge, especially in Lesotho, about how the procedure is performed. Additional empirical evidence can help inform government, international organizations, NGOs, and traditional healers about the associated risks of including TMC in policies of mass male circumcision. If TMC can be made as safe and medically effective as VMMC, it could be used as an efficacious element of a more comprehensive HIV prevention package in several developing countries.

## Supporting information

S1 Table Male circumcision and risky sexual behavior. Association between male circumcision and measures of risky sexual behavior.
(PDF)
