# Supplementary material for: Is traditional male circumcision effective as an HIV prevention strategy? Evidence from Lesotho
Source: PLoS One. 2017 May 12;12(5):e0177076. doi: 10.1371/journal.pone.0177076 (PMC5428932; doi:10.1371/journal.pone.0177076)
Supplement: S1 Table — Association between male circumcision and measures of risky sexual behavior. (PDF) [file pone.0177076.s001.pdf]

Supporting Information

S1 Table. Male circumcision and risky sexual behavior. Association between male circumcision and measures of risky sexual behavior.

S1 Table. Male circumcision and risky sexual behavior

Dependent Variable:	Age first marriage	Age first intercourse	No condom last intercourse	Extramarital relationships	Sexually active last 4 weeks	Transactional sex	Alcohol last sex	No. people had sex last year	No. partners in lifetime
	(1)	(2)	(3)	(4)	(5)	(6)	(7)	(8)	(9)
Circumcised	0.0101	-0.0150	0.0817	-0.0160	0.1182**	-0.0076	-0.0076	0.0585	0.1520
	(0.020)	(0.016)	(0.052)	(0.065)	(0.052)	(0.016)	(0.030)	(0.058)	(0.143)
Circumcised X TMC	-0.0484**	0.0078	-0.0029	0.1035	-0.0993*	0.0115	0.0191	0.0805	0.0046
	(0.020)	(0.016)	(0.055)	(0.068)	(0.054)	(0.017)	(0.032)	(0.065)	(0.152)
Age	0.0303***	0.0234***	-0.0099	-0.0053	0.0218***	0.0023	0.0120***	0.0165	0.0904***
	(0.003)	(0.002)	(0.008)	(0.009)	(0.006)	(0.002)	(0.003)	(0.012)	(0.020)
Age squared	-0.0003***	-0.0002***	0.0002*	0.0000	-0.0003***	-0.0000	-0.0001***	-0.0003**	-0.0008***
	(0.000)	(0.000)	(0.000)	(0.000)	(0.000)	(0.000)	(0.000)	(0.000)	(0.000)
Educ secondary or higher	0.0230*	0.0049	-0.1621***	-0.0070	0.0117	-0.0043	0.0005	0.1239***	0.2014**
	(0.012)	(0.008)	(0.028)	(0.036)	(0.027)	(0.007)	(0.014)	(0.046)	(0.088)
Rural area	-0.0186	0.0366***	0.0688**	0.0554	-0.0605*	-0.0063	-0.0192	0.0026	-0.1609*
	(0.015)	(0.009)	(0.032)	(0.040)	(0.032)	(0.008)	(0.017)	(0.051)	(0.097)
Married	0.0092	0.0071	0.3014***		0.4055***	-0.0186***	-0.0260**	0.0797	-0.0422
	(0.015)	(0.010)	(0.029)		(0.026)	(0.006)	(0.012)	(0.051)	(0.094)
Poor	0.0208*	-0.0021	0.1223***	-0.0366	-0.0127	0.0058	0.0155	-0.0316	-0.0443
	(0.012)	(0.009)	(0.032)	(0.035)	(0.029)	(0.007)	(0.013)	(0.036)	(0.081)
Self-employed in agriculture	-0.0058	-0.0062	0.0243	0.0028	-0.0327	-0.0063	0.0052	0.0128	-0.1373*
	(0.011)	(0.008)	(0.027)	(0.028)	(0.026)	(0.006)	(0.012)	(0.043)	(0.077)
Working yearly	0.0165*	0.0030	0.0520*	0.0890***	0.0211	0.0011	-0.0104	-0.0041	0.0114
	(0.010)	(0.008)	(0.027)	(0.028)	(0.025)	(0.006)	(0.012)	(0.034)	(0.064)
Household size	-0.0023	-0.0000	0.0109**	-0.0144***	0.0006	0.0006	-0.0007	-0.0062	-0.0106
	(0.002)	(0.001)	(0.004)	(0.005)	(0.004)	(0.001)	(0.002)	(0.005)	(0.013)
No. times away in past year	-0.0004	-0.0009	0.0011	0.0023	0.0010	-0.0004	0.0009	0.0079**	0.0056
	(0.001)	(0.001)	(0.002)	(0.002)	(0.002)	(0.001)	(0.001)	(0.004)	(0.006)
Away in past month	-0.0013	0.0087	-0.0135	-0.0236	0.0536*	-0.0018	-0.0141	0.0121	-0.0000
	(0.011)	(0.008)	(0.028)	(0.034)	(0.028)	(0.007)	(0.013)	(0.039)	(0.075)
District FE	✓	✓	✓	✓		✓	✓	✓	✓
Religion controls	✓	✓	✓	✓	✓	✓	✓	✓	✓
Model	Poisson	Poisson	Logit (ME)	Logit (ME)	Logit (ME)	Logit (ME)	Logit (ME)	Poisson	Poisson
Mean Dep Var	23.76	17.71	0.440	0.293	0.508	0.0269	0.0927	1.185	7.771
Observations	1,469	2,711	2,341	1,282	2,672	2,640	2,662	2,672	2,575

*** p<0.01, ** p<0.05, * p<0.1. Standard errors in parentheses. The main specification is an Ordinary Least Square (OLS). Religion controls include dummies for whether the respondent is Catholic, Protestant or Islamic. District fixed effects are also included. Standard errors are clustered at the smallest enumeration area.
